# From Style to Wellness: How Fashion Influencers Are Affecting Healthy Behaviors in Saudi Arabia

**DOI:** 10.7759/cureus.57749

**Published:** 2024-04-07

**Authors:** Abdulrahman M Albeshry, Najim Z Alshahrani, Mohamed Baklola, Mohamed Terra

**Affiliations:** 1 Department of Family and Community Medicine, Faculty of Medicine, University of Jeddah, Jeddah, SAU; 2 Department of Public Health, Mansoura University, Mansoura, EGY

**Keywords:** healthy behavior, social media platforms, influencers, youtube influencers, media literacy, digital health promotion, fashion influencers, saudi arabia, health behaviors, social media influencers

## Abstract

Background

Social media influencers, particularly those in the fashion domain, have become prominent sources of health-related information, influencing the health behaviors of their followers, especially within the unique socio-cultural context of Saudi Arabia. This study aims to examine the relationship between following fashion influencers on social media and the adoption of healthy behaviors among Saudi residents.

Methods

A descriptive cross-sectional study with an analytic component was conducted from August 25, 2023, to December 15, 2023, among Saudi residents aged 18 years and older who actively follow fashion influencers. Data were collected via a structured, validated questionnaire distributed through various social media platforms. Data analysis was performed by employing descriptive statistics, Pearson's chi-square test, and logistic regression analyses to identify predictors of negative health behavior outcomes.

Results

The study included 466 participants, revealing that despite insignificant differences in age and gender, postgraduate education was associated with negative outcomes (p = 0.016). Notably, 8.7% of individuals with negative outcomes held postgraduate degrees, compared to 2.9% in the non-negative group. Participants with negative outcomes more frequently followed influencers offering health tips (p = 0.01) and advocating a healthy lifestyle (p < 0.001). Logistic regression identified individuals who followed influencers with fewer than 10K followers had significantly higher odds of negative outcomes (OR = 3.6, 95% CI: 1.4-8.9, p = 0.006).

Conclusion

Following fashion influencers on social media can influence health behaviors among Saudis, with both positive and negative outcomes. Critical evaluation of influencer content is crucial to avoid potential adverse effects. Further research is also needed to explore the dynamics of influencer impact on health outcomes and to develop strategies for effective health communication in the digital age.

## Introduction

Social media influencers (SMIs) have emerged as a significant alternative source of health-related information for people. They are characterized as individuals who accumulate substantial followings on social media platforms and wield considerable influence over their audience through captivating content. SMIs play a pivotal role in shaping perceptions and behaviors [1]. On the one hand, they serve as authentic sources and positive role models, offering valuable insights into critical health topics like nutrition and mental well-being [1,2].

Consequently, SMIs can disseminate constructive public health messages to people, a demographic often challenging to reach via traditional communication channels such as television, newspapers, or radio [3,4]. Conversely, SMIs may lack formal health expertise and may prioritize commercial interests, potentially endorsing or advertising unhealthy products [5,6].

This dilemma raises concerns regarding the suitability of SMIs as alternatives to traditional information sources, such as direct consultation with healthcare professionals like doctors, nutrition experts, psychologists, or parents [7,8]. Consequently, it remains ambiguous whether SMIs contribute positively or impede the health outcomes of populations who confront pressing challenges such as escalating rates of obesity and mental health issues [9].

An influencer, within the context of social media, is a term denoting an individual who possesses the capacity to significantly influence their followers' beliefs, behaviors, habits, or purchasing decisions [10]. In the realm of social media, a "follower" refers to a user who subscribes to a specific account or individual to access more of their content on the platform. Influencers typically command a large following, granting them the potential to effectuate widespread behavioral changes. These individuals often specialize in a particular niche and regularly post content related to their area of expertise, sometimes unrelated to their specialty [10]. For instance, James Charles, a prominent figure in the beauty industry, exemplifies a fashion influencer with 22.7 million Instagram followers and 23.9 million subscribers on YouTube [11]. Through his platform, James Charles frequently showcases and reviews various makeup products, potentially shaping the perceptions of his audience toward these products. Beyond solely impacting purchasing decisions, influencers also exert considerable influence over the beliefs, behaviors, and adoption of healthy lifestyles among their followers.

This behavior change may indeed stem from the multifaceted influence of fashion influencers. Followers might find motivation to purchase products, such as makeup or specific video games, based on positive endorsements from influencers. Furthermore, they may feel encouraged to adopt behaviors outside of their usual routines, such as engaging in exercise or modifying their lifestyle choices, as promoted or demonstrated by these influencers. Such shifts in behavior can have implications for their overall health and well-being.

In Saudi Arabia, the intersection of health, social media, and cultural dynamics presents a unique landscape [12]. The country's young population, high smartphone penetration, and evolving social norms make it a fertile ground for digital health promotion. Fashion influencers, many of whom are Saudis, resonate with the local audience by blending health advice with cultural relevance. They not only offered fashion tips but also represented a lifestyle aspiration within the socio-cultural context of Saudi Arabia. However, the impact of following fashion influencers on health behaviors in Saudi Arabia remains under-explored. While global studies provide insights, the unique cultural, social, and economic fabric of Saudi Arabia necessitates a localized understanding.

The study aims to analyze predictors of negative health behaviors among Saudi residents influenced by fashion influencers on social media, examining demographic, socio-economic, and influencer-related factors while exploring Saudis' perceptions of influencer credibility and impact on health choices. This research is timely amidst Saudi Arabia's digital transformation and growing focus on public health and wellness.

## Materials and methods

Study design and study period

From August 25, 2023, through December 15, 2023, a descriptive cross-sectional study with an analytical component was carried out among the general population of Saudi Arabia. The study included Saudi residents aged 18 years and older who actively follow fashion influencers on social media platforms.

Sample size

We used the Raosoft sample size calculator (Raosoft, Inc., Seattle, WA) to determine the minimum number of participants needed for our study [13]. To obtain accurate results, we assumed a population proportion of 50%, a margin of error of 5%, and a confidence interval of 95%. The calculator indicated that we would need at least 385 participants.

Sampling and data collection approach

In our study, we employed a community-based convenience sampling method to select participants. The target population comprised individuals residing in Saudi Arabia who follow one or more fashion influencers on social media platforms such as X "formerly Twitter," WhatsApp, and Facebook. Crucially, we focused on those who not only follow these influencers but also actively engage with their content, indicated by actions like liking, commenting, or sharing posts, at least once a week. This engagement criterion was established to ensure that the participants had a significant level of interaction with fashion influencer content, which is central to our study's aim.

Data were collected using an anonymous online questionnaire. The questionnaire had four parts. The first part asked for socio-demographic information, such as where you live, your gender, age, education level, employment status, and how much you earn each month. The second section assessed health-related characteristics. The third section focused on the characteristics of fashion influencers, and the fourth section delved into the perception and impact of influencers on health attitudes and behaviors (Appendix).

The development of the questionnaire involved an extensive review of the relevant literature, followed by pre-testing with a sample of 30 participants from Saudi Arabia. Additionally, four experts with expertise in public health and sociology evaluated the survey for clarity and reliability. The pilot study's internal consistency was assessed using Cronbach’s α reliability coefficient, which indicated moderate to good reliability (Cronbach’s α = 0.72). The questionnaire design process was thus carefully executed to ensure that the final product would be reliable and valid for use in future research.

Statistical analysis

Statistical Package for Social Sciences (SPSS) version 25 for Windows (IBM Corp., Armonk, NY) was used for data analysis. Descriptive statistics were used to summarize the demographic characteristics of the participants, including percentages, frequencies, means, and standard deviations. To compare categorical variables between groups, Pearson's chi-square test was used.

Additionally, logistic regression was utilized to examine the relationship between following fashion influencers and experiencing negative results among participants. Both univariate and multivariate logistic regression analyses were performed. The univariate analysis identified individual factors significantly associated with experiencing negative results, while the multivariate analysis adjusted for potential confounders and provided a more comprehensive understanding of the independent predictors of health behavior changes. Odds ratios (ORs) with 95% confidence intervals (CIs) were calculated to estimate the strength and direction of associations. A p-value below 0.05 was considered statistically significant in all analyses.

## Results

Socio-demographic and health-related characteristics

The study investigated the socio-demographic and health-related characteristics of 466 Saudi residents who follow fashion influencers, analyzing their influence on health behaviors. Our analysis primarily focused on identifying negative outcomes associated with this engagement.

Among the participants, the mean age was 26 years (SD ±8) for those without negative health experiences and 27 years (SD ±9) for those with negative outcomes, a difference that was not statistically significant (p = 0.18). Females constituted the majority in both groups, 86.2% in the non-negative and 82.5% in the negative outcome groups, with no significant gender-based variation in outcomes (p = 0.29).

Educational attainment showed a significant correlation with health outcomes. Participants with postgraduate degrees were more likely to experience negative health behaviors, with 8.7% in the negative outcome group compared to 2.9% in the non-negative group (p = 0.016). The employment sector, however, did not show a significant differentiation in health outcomes (p = 0.12), even though a slightly higher proportion of medical professionals were observed in the negative outcome group.

No significant differences were found in monthly family income, BMI categories, smoking status, physical activity levels, or dietary habits concerning negative health outcomes (p-values ranging from 0.10 to 0.79). Nonetheless, a trend toward poorer health perception was noted among participants experiencing negative outcomes, with 13.7% rating their health as bad, compared to 7.8% in the non-negative group, although this did not reach statistical significance (p = 0.12).

The detailed demographic and health-related characteristics, segmented by the participants' experiences with fashion influencers, are summarized in Table 1.

**Table 1:** Socio-demographic and health-related characteristics of participants, stratified by negative outcomes from adopting fashion influencers and their health habits. * Statistically significant (p < 0.05).

Variable		Did not experience negative results (N = 283)	Experienced negative results (N = 183)	P-value
Age (mean ± SD)		26 (8)	27 (9)	0.18
Gender	Female	244 (86.2)	151 (82.5)	0.29
	Male	39 (13.8)	32 (17.5)	
Marital status	Single	181 (64)	114 (62.3)	0.69
	Married	102 (36)	69 (37.7)	
Residence	Southern region	56 (19.8)	41 (22.4)	0.30
	Eastern region	80 (28.3)	37 (20.2)	
	Northern region	26 (9.2)	19 (10.4)	
	Western region	69 (24.4)	55 (30.1)	
	Central region	52 (18.4)	31 (16.9)	
Educational level	Secondary school	98 (34.6)	54 (29.5)	0.01^*^
	Bachelor’s degree	177 (62.5)	113 (61.8)	
	Postgraduate degree	8 (2.9)	16 (8.7)	
Field of work	Medical	43 (15.2)	38 (20.8)	0.12
	Non-medical	39 (13.8)	34 (18.6)	
	Student	135 (47.7)	79 (43.2)	
	Not employed	66 (23.3)	32 (17.5)	
Monthly family income (Saudi Riyal)	Less than 3,000	38 (13.4)	31 (16.9)	0.79
	3,001- 10,000	62 (21.9)	38 (20.8)	
	10,001-20,000	127 (44.9)	79 (43.2)	
	More than 20,000	56 (19.8)	35 (19.1)	
Body mass index (BMI)	Underweight	45 (15.9)	34 (18.6)	0.51
	Normal weight	139 (49.1)	97 (53)	
	Overweight	73 (25.8)	38 (20.8)	
	‎Obese	26 (9.2)	14 (7.7)	
Current smoker	No	242 (85.5)	147 (80.3)	0.16
	Yes	41 (14.5)	36 (19.7)	
Frequency of physical activity/week	No physical activity	138 (48.8)	83 (45.4)	0.10
	Once	68 (24)	32 (17.5)	
	Two to three times	44 (15.5)	41 (22.4)	
	More than three times	33 (11.7)	27 (14.8)	
Following healthy diet	No	182 (64.3)	113 (61.7)	0.62
	Yes	101 (35.7)	70 (38.3)	
How do you describe your health status in general?	Bad	22 (7.8)	25 (13.7)	0.12
	Good	146 (51.6)	91 (49.7)	
	Excellent	115 (40.6)	67 (36.6)	

Characteristics of fashion influencers followed

Analysis of fashion influencers followed by participants revealed subtle differences. While the gender of influencers followed showed slight variation, it did not reach statistical significance (p = 0.10). The average number of influencers followed and their approximate average number of followers did not significantly differ between groups (p = 0.51 and p = 0.03, respectively). However, participants with negative outcomes more frequently followed influencers offering health tips (p = 0.01) and promoting a healthy lifestyle (p < 0.001) (Table 2).

**Table 2:** Characteristics of fashion influencers followed, stratified by negative outcomes from adopting influencers and their health habits. * Statistically significant (p < 0.05).

Characteristics/variables		Did not experience negative results (N = 283)	Experienced negative results (N = 183)	P-value
Gender of influencers followed	Female	68 (24)	49 (26.8)	0.10
	Male	16 (5.7)	19 (10.4)	
	Both	199 (70.3)	115 (62.8)	
Average number of influencers followed	<10	106 (37.5)	64 (35)	0.51
	10-50	115 (40.6)	74 (40.4)	
	50-100	37 (13.1)	21 (11.5)	
	100+	25 (8.8)	24 (13.1)	
Approximate average number of followers of influencers followed	Don’t know	149 (52.7)	73 (39.9)	0.03*
	<10K	8 (2.8)	14 (7.7)	
	10K-100K	34 (12)	27 (14.8)	
	100K-1M	57 (20.1)	40 (21.9)	
	>1M	35 (12.4)	29 (15.8)	
Do the influencers on social media whom you specifically choose to follow provide health tips on their platforms?	No	33 (11.7)	21 (11.5)	0.01*
	Some of them	219 (77.4)	133 (72.7)	
	Neutral	21 (7.4)	9 (4.9)	
	Yes, all of them	10 (3.5)	20 (10.9)	
Do the influencers you follow on social media promote a daily healthy lifestyle?	No	15 (5.3)	11 (6)	<0.01*
	Some of them	220 (77.7)	122 (66.7)	
	Neutral	42 (14.8)	29 (15.8)	
	Yes, all of them	6 (2.1)	21 (11.5)	

Perception and impact of influencers on health attitudes and behaviors

Regarding perceptions of health advice from influencers, most participants believed influencers only sometimes adhered to their recommendations. Trust in such advice from non-healthcare professionals was conditional, with most respondents sometimes trusting it. Skepticism toward influencers' health advice and perceived motivations were associated with a reduced risk of negative outcomes, highlighting the protective effect of critical engagement with influencer content, as shown in Table 3.

**Table 3:** Perception and impact of influencers on health attitudes and behaviors.

Variable		Frequency	%
Do social media influencers adhere to the health advice they provide to the public?	Always	41	8.8%
	Sometimes	414	88.8%
	Never	11	2.4%
Do you trust health advice given by influencers on social media who are not in the healthcare field?	Always	25	5.4%
	Sometimes	388	83.3%
	Never	53	11.4%
Do you think promoting healthy products by social media influencers is solely for monetary purposes?	Sometimes	333	71.5%
	Never	26	5.6%
	Always	107	23.0%
Have you noticed that influencers who are committed to proper healthy habits tend to have good health?	Some of them	244	52.4%
	No	12	2.6%
	Yes	210	45.1%
Do you agree that social media influencers accurately represent a healthy lifestyle?	Some of them	340	73.0%
	No	73	15.7%
	Yes	53	11.4%
Overall, to what extent are you satisfied with the changes that have occurred in your lifestyle due to the influence of celebrities on social media?	Completely satisfied	86	18.5%
	Somewhat satisfied	315	67.6%
	Not satisfied	65	13.9%

Predictors of negative health outcomes linked to fashion influencer-endorsed health habits

Binary logistic regression analysis revealed significant predictors of negative outcomes resulting from adopting health habits endorsed by fashion influencers. Educational level emerged as a noteworthy predictor, with postgraduates exhibiting increased odds of reporting negative outcomes (OR = 3.2, p = 0.02). Moreover, influencer characteristics, including the average number of followers and the promotion of health-related content such as tips and healthy lifestyle advocacy, significantly impacted the likelihood of negative outcomes. Notably, skepticism toward influencers' health advice and perceived promotional motivations were associated with a diminished risk of negative outcomes, highlighting the importance of critically engaging with influencer content to mitigate potential adverse effects (Table 4).

**Table 4:** Binary logistic regression analysis of experiencing negative results regarding different variables. * Statistically significant (p < 0.05). OR = odds ratio; r = reference.

Variable		Univariate regression		Multivariate regression	
		OR (95% CI)	P-value	OR (95% CI)	P-value
Demographic factors					
Educational level	Secondary school	1 (r)			
	Bachelor’s degree	1.6 (0.8-1.7)	0.479		
	Postgraduate degree	3.6 (1.5-9)	0.006*	3.2 (1.2-8.8)	0.02*
Influencer characteristics					
Approximate average number of followers of influencers followed	Don’t know	1 (r)			
	<10K	3.6 (1.4-8.9)	0.006*	3.1 (1.1-8.3)	0.03*
	10K-100K	1.6 (0.9-2.8)	0.10		
	100K-1M	1.4 (0.9-2.3)	0.15		
	>1M	1.7 (0.9-2.9)	0.06		
Do the influencers on social media whom you specifically choose to follow provide health tips on their platforms?	No	1 (r)			
	Some of them	0.9 (0.5-1.7)	0.87		
	Neutral	0.7 (0.2-1.7)	0.41		
	Yes, all of them	3.1 (1.2-8)	0.01*		
Do the influencers you follow on social media promote a daily healthy lifestyle?	No	1 (r)			
	Some of them	1.3 (0.6-3)	0.498		
	Neutral	1.5 (0.7-2.1)	0.411		
	Yes, all of them	6.3 (2.5-16.1)	0.001*		
Other factors					
Do social media influencers adhere to the health advice they provide to the public?	Always	1 (r)			
	Sometimes	0.4 (0.2-0.7)	0.003*		
	Never	1.7 (0.4-7.4)	0.4		
Do you trust health advice given by influencers on social media who are not in the healthcare field?*	Always	1 (r)			
	Sometimes	0.1 (0.05-0.4)	0.001*	0.2 (0.07-0.6)	0.007*
	Never	0.2 (0.06-0.6)	0.006*	0.3 (0.08-0.9)	0.04*
Do you think that the promotion of healthy products by social media influencers is solely for monetary purposes?	Always	1 (r)			
	Sometimes	0.5 (0.4-0.8)	0.006*		
	Never	1.4 (0.6-3.3)	0.45		
Do you agree that social media influencers accurately represent a healthy lifestyle?	No	1 (r)			
	Yes	1.2 (0.6-2.3)	0.69		
	Some of them	0.6 (0.3-0.9)	0.02*	0.6 (0.3-0.9)	0.04*

Main sources and platforms for health habits information

**Figure 1:**
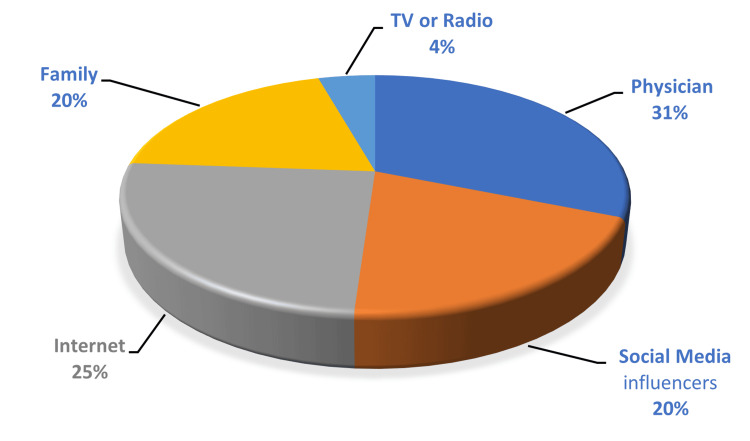
The main source of learning and following healthy habits.

**Figure 2:**
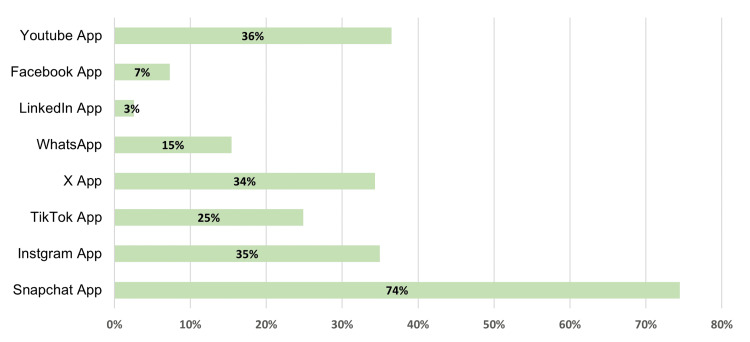
Social media platforms are used to search for information about healthy habits.

The primary sources for learning and following healthy habits were identified as physicians, internet searches, and influencers on social media. The most used social media platforms for searching for information about healthy habits were Snapchat, YouTube, Instagram, and X (Figures 1, 2).

## Discussion

In our study, we discovered that following fashion influencers on social media has a nuanced impact on health behaviors among Saudi residents, with educational attainment notably influencing negative health outcomes. The findings underscore the complex relationship between social media influence and health-related decisions, emphasizing the need for tailored health promotion strategies. By understanding these dynamics, health professionals can develop interventions that address the specific ways in which fashion influencers affect public health perceptions and behaviors, thereby fostering healthier lifestyle choices within the community.

Our study participants' demographic and health-related characteristics reveal a predominantly young and female audience engaged with fashion influencers, aligning with global trends in social media usage and health information-seeking behavior [14,15]. Notably, the lack of significant differences in socio-demographic variables like age, marital status, and geographical region between those experiencing negative outcomes from influencer engagement and those who do not, suggests that the impact of influencers transcends these basic demographic lines. This indicates the broad influence of social media across different segments of the Saudi population [16].

Our study uniquely highlights the role of educational attainment in modulating the influence of fashion influencers. Participants with postgraduate degrees were more likely to report negative outcomes from adopting health behaviors promoted by influencers. This finding could suggest that higher education levels may correlate with greater engagement with influencer content, potentially leading to higher scrutiny and, consequently, a more critical assessment of the outcomes [17]. Alternatively, it may reflect a higher awareness or willingness among more educated individuals to attribute health behavior outcomes to their sources, including influencers [18].

The characteristics of fashion influencers followed by participants, particularly those promoting health tips and healthy lifestyles, were significantly associated with negative outcomes. This association underscores the complexity of influencer impact, where the promotion of ostensibly positive health behaviors does not uniformly translate into beneficial outcomes for followers. It highlights the need for critical evaluation of health content on social media, echoing concerns about the accuracy and reliability of health information disseminated by non-experts [19,20].

The study's findings on perception and impact underscore the conditional trust placed in health advice from influencers. Most participants exhibited skepticism toward health recommendations from influencers, associating this critical engagement with a reduced risk of negative outcomes. This suggests that while influencers can play a role in health communication, their influence is moderated by the audience's ability to critically assess the information presented. This critical engagement acts as a protective factor, mitigating potential negative impacts of influencer-promoted health behaviors [10,20].

The nuanced relationship between influencer characteristics, follower engagement, and health outcomes highlights the potential of influencers as tools for public health promotion, provided their reach is leveraged judiciously [21]. Our findings suggest that influencers who promote health tips and healthy lifestyles can influence health behaviors, but the impact varies based on the audience's critical engagement with the content. This underscores the importance of enhancing media literacy among the public to foster a more discerning consumption of health information on social media.

Moreover, the significant role of educational attainment in influencing health behavior outcomes points to the need for targeted health communication strategies that consider the audience's educational background. Tailoring messages to different educational levels could improve the effectiveness of health promotion efforts on social media [22].

Future research should explore the mechanisms through which critical engagement with influencer content protects against negative outcomes. Understanding these processes could inform the development of interventions to enhance media literacy and critical thinking about health information on social media [23]. Additionally, studies should investigate the influence of influencers across a broader range of health behaviors and outcomes, including positive impacts, to fully understand the potential of influencers in health promotion.

Research examining the role of cultural and social norms' role in mediating fashion influencers' influence on health behaviors in Saudi Arabia would also provide valuable insights. Such studies could help tailor health communication strategies to Saudi audiences' unique cultural context, maximizing influencers' positive impact on public health [24].

This study's strength lies in its focused examination of the impact of fashion influencers on health behaviors within the Saudi Arabian context, an area yet to be explored in the literature. Utilizing a comprehensive cross-sectional design, the research captured a wide range of demographic and socio-economic factors, offering a detailed insight into the nuances of social media influence. The large sample size and the use of validated questionnaires enhanced the reliability and validity of the findings, making a significant contribution to understanding the role of digital influencers in shaping health-related attitudes and behaviors in a culturally specific setting.

This study has several limitations. Firstly, its cross-sectional design precludes the establishment of causality between following fashion influences and changes in health behaviors. Secondly, the reliance on self-reported data may introduce biases, including recall bias and social desirability bias, potentially affecting the accuracy of the reported behaviors and perceptions. Additionally, the sample, drawn from an online survey, might not fully represent the Saudi population, particularly under-represented groups who may have limited access to the internet or lower social media engagement. Furthermore, the study focuses primarily on negative outcomes associated with following fashion influencers, potentially overlooking positive health behavior changes inspired by influencer engagement. Lastly, the rapidly evolving nature of social media platforms and influencer dynamics means that the findings may have limited temporal relevance, necessitating ongoing research to capture these changes.

## Conclusions

This study sheds light on the complex relationship between fashion influencers and the adoption of healthy behaviors in Saudi Arabia, revealing both the potential and challenges of leveraging social media for health promotion. It underscores the significance of critical media literacy in mitigating the risks associated with influencer-led health advice, highlighting the conditional trust that followers place in such information. Importantly, the findings call for a nuanced approach to public health strategies, suggesting that effective health communication in the digital age requires collaboration between public health entities and influencers to ensure the dissemination of accurate and beneficial health information. As the digital landscape continues to evolve, ongoing research and adaptive public health interventions will be crucial in maximizing the positive impact of social media on public health outcomes, especially within the unique socio-cultural context of Saudi Arabia.

## Appendices

From elegance to wellness: The impact of fashion influencers on health behaviors in Saudi Arabia

Welcome to our study exploring how fashion influencers affect health behaviors in Saudi Arabia. We aim to understand the dynamics between adopting lifestyles promoted by fashion influencers and the consequent health effects.

Participants in this study are from various ages, social, and economic backgrounds, living in different regions of the Kingdom.

Your responses will help us analyze the relationship between following fashion influencers and overall health status, and to understand how their guidance can positively or negatively impact health. Thank you for your participation and contribution to this important study.

Section One: Demographic and Health Characteristics

1. Age

• Years

2. Gender

• Male

• Female

3. Marital status

• Single

• Married

4. Place of residence (region)

• Southern

• Eastern

• Northern

• Western

• Central

5. Educational level

• High school

• Bachelor’s degree

• Master’s/Ph.D.

6. Field of work

• Medical

• Non-medical

• Student

• Unemployed

7. Monthly household income (Saudi riyal)

• <3,000

• 3,001-10,000

• 10,001-20,000

• >20,000

8. Body mass index (BMI)

• Underweight

• Normal weight

• Overweight

• Obesity

9. Are you currently a smoker?

• Yes

• No

10. Physical activity weekly

• None

• Once

• 2-3 times

• >3 times

11. Do you follow a healthy diet?

• Yes

• No

12. Overall health condition

• Poor

• Good

• Excellent

Section Two: Characteristics of Fashion Influencers

1. Gender of influencers you follow

• Female

• Male

• Both

2. The average number of influencers you follow

• <10

• 10-50

• 50-100

• >100

3. Followers of the influencers you follow

• Don’t know

• <10,000

• 10,000-100,000

• 100,000-1 million

• >1 million

4. Health tips provided by influencers

• Yes, all

• Some

• No

5. Promotion of a healthy lifestyle

• Yes, all

• Some

• No

Section Three: Perceptions and Influence of Influencers on Health Trends and Behaviors

1. Adherence of influencers to their health advice

• Always

• Sometimes

• Never

2. Trust in non-medical influencers’ health advice

• Always

• Sometimes

• Never

3. Promotion of health products for financial reasons

• Always

• Sometimes

• Never

4. Influencers with good health habits have good health

• Yes

• Some

• No

5. Accurate representation of a healthy lifestyle by influencers

• Yes

• Some

• No

6. Satisfaction with lifestyle changes due to influencers’ impact

• Completely satisfied

• Somewhat satisfied

• Not satisfied

Instructions for Completing the Survey

Please take your time to read each question carefully and mark your response as accurately as possible. Your participation is voluntary, and you may choose not to answer any specific question. Your responses will be kept confidential and used solely for research purposes.

How to Submit

After completing the survey, please check your answers to ensure they accurately reflect your views and experiences. Once satisfied, follow the submission instructions provided by the survey platform or organization conducting this study. Typically, this will involve clicking a "Submit" button at the end of the survey or sending your responses to a specified email address or online portal.

Consent

By submitting your responses, you consent to the use of your data as part of this research study. The information collected will contribute to our understanding of the influence of fashion influencers on health behaviors in Saudi Arabia.

Thank you. We appreciate your time and effort to participate in this study. Your insights are valuable to us and will contribute significantly to our research. If you have any questions about the survey or the study, please contact the research team using the contact information provided.
